# Therapeutic mRNA vaccines for lung cancer: reshaping the tumor microenvironment to enhance anti-tumor immunity

**DOI:** 10.3389/fimmu.2026.1916788

**Published:** 2026-08-25

**Authors:** Hao Wu, Ruiqi Weng, Siyi Gu, Jiyuan Wang, Xiaoyan Ma, Jiaxuan Li, Xiaotian Tie, Chongmin Huang, Keda Chen

**Affiliations:** 1 Key Laboratory of Artificial Organs and Computational Medicine in Zhejiang Province, Shulan International Medical College, Zhejiang Shuren University, Hangzhou, China; 2 Zhejiang Chinese Medical University, Hangzhou, China; 3 School of Basic Medicine and Forensic Medicine, Hangzhou Medical College, Hangzhou, China

**Keywords:** immunotherapy, lung cancer, mRNA vaccine, tumor antigens, tumor microenvironment

## Abstract

Traditional treatments for lung cancer include surgery, radiotherapy, chemotherapy, and targeted therapies (such as Erlotinib, Gefitinib, and Crizotinib), as well as immunotherapy (such as Nivolumab and Pembrolizumab). However, these approaches face challenges such as drug resistance, limited efficacy, and significant side effects, making it difficult to improve long-term prognosis. Therefore, tumor vaccines, as a novel immunotherapy strategy, have become a focus of research, aiming to precisely activate anti-tumor immune responses and address the shortcomings of existing treatments. In recent years, mRNA vaccine technology has developed rapidly, especially following the successful application of COVID-19 vaccines, demonstrating its immense potential for rapid development and large-scale production. mRNA cancer vaccines induce durable anti-tumor immune responses and reshape the tumor microenvironment by delivering mRNA encoding tumor-specific antigens, enabling targeted therapy. Through the dual synergistic pathways of direct local modulation by the vector/encoded factors and secondary effects mediated by antigen-specific T-cell activation, the tumor microenvironment is reshaped, thereby inducing durable antitumor immune responses. Against this background, this review systematically evaluates the current status and prospects of mRNA vaccines in lung cancer treatment, with a focus on their progress in precisely modulating the tumor microenvironment, identifying novel tumor antigens, discovering immune biomarkers, and related clinical studies. Additionally, the review discusses the process optimization of lipid nanoparticle (LNP) delivery systems, aiming to provide theoretical support and practical guidance for the development of safe and effective therapeutic vaccine platforms for lung cancer.

## 1 Introduction

Lung cancer is one of the leading causes of cancer-related deaths and is generally classified into small cell lung cancer (SCLC) and non-small cell lung cancer (NSCLC) types (1–3). Epidemiological surveys indicate that lung cancer remains a major global health burden. According to the GLOBOCAN 2022 estimates, approximately 2.48 million new lung cancer cases were reported worldwide in 2022, representing an increase of approximately 280000 cases compared with the estimated number in 2020 and accounting for 12.4% of all newly diagnosed cancers (2, 4, 5). Currently, NSCLC accounts for 85% or more of all lung cancer cases. The histological subtypes include lung adenocarcinoma (LUAD) and squamous cell carcinoma (LUSC), which together constitute a significant proportion of NSCLC cases, accounting for 40% and 25%, respectively (2). In LUAD, the most common activating mutation is the *KRAS*^G12C^ mutation, which is prevalent in adenocarcinoma (37.2%) compared to squamous cell carcinoma (4.4%) (6). This mutation spectrum suggests that *KRAS*^G12C^ is a critical molecular marker and therapeutic target for LUAD, providing important insights into the molecular subtyping of lung cancer and the optimization of targeted therapy strategies (7). Although *KRAS*^G12C^ is one of the most representative key driver mutations in lung cancer, with significant impacts on tumorigenesis and treatment, other mutations such as *KRAS*^G12D^ and *KRAS*^G12V^ also play important biological roles in LUAD. This phenomenon suggests that the complex interactions between genetic factors not only influence patient prognosis and sensitivity to targeted and immunotherapy, but also provide important potential targets for designing mRNA therapeutic vaccines for lung cancer aimed at specific mutation subtypes (8). At present, mutation-specific small-molecule inhibitors targeting *KRAS* have been developed. For example, Adagrasib (MRTX849) irreversibly binds to the inactive state of *KRAS*^G12C^ and exerts selective covalent inhibition (9). Beyond gene-targeted therapeutics, first-line treatment for non–small cell lung cancer (NSCLC) still relies predominantly on immunotherapy-based combination regimens, most commonly pairing programmed cell death receptor 1/programmed cell death ligand 1 (PD-(L)1) blockade with cytotoxic chemotherapy. Among these, PD-1 and cytotoxic T-lymphocyte–associated antigen 4 (CTLA-4) are two complementary and non-redundant co-inhibitory receptor targets that regulate T-cell responses. Immune checkpoint inhibitors (ICIs) developed against these pathways can block the interaction of CTLA-4 and the PD-1/PD-L1 axis with their respective ligands, thereby enabling effective treatment of lung cancer (5, 10).

However, the objective response rate (ORR) to immune checkpoint inhibitor monotherapy remains modest across most solid tumors, typically ranging from 10% to 30%. Moreover, sensitive and specific predictive biomarkers are still lacking, limiting accurate patient stratification and potentially resulting in missed or inappropriate treatment selection. Even among patients with high PD-L1 expression and elevated tumor mutational burden, a substantial proportion exhibit suboptimal responses to current immunotherapy regimens, making it difficult to achieve optimal clinical benefit (11). Studies suggest that patients with lung cancer may be at a higher risk of developing immune-related adverse events (irAEs), a pattern that may be associated with the respiratory toxicity of ICIs. For example, in NSCLC patients treated with the newer immunotherapeutic agent cemiplimab, cemiplimab-associated irAEs occurred in approximately 50% of patients and were reported to be more severe than those observed in other solid tumors (12).

mRNA vaccines, as an emerging immunotherapeutic strategy, have made significant strides in the field of infectious diseases in recent years. Particularly in the application of COVID-19 vaccines, they have fully demonstrated their potential for rapid development and the induction of potent immune responses (13). Notably, the mRNA platform is not limited to simple antigen expression. It can also synergistically reshape adaptive immunity by activating innate immune sensing and antigen presentation (14). Its unique mechanistic advantages and high tunability lay an important foundation for the development of therapeutic cancer vaccines that efficiently induce T-cell responses. Building upon this foundation, mRNA technology is accelerating its expansion into the field of cancer therapy, gradually emerging as one of the key focal points in both fundamental and clinical research. Although therapeutic mRNA vaccines against tumors remain in the early stages of development, their potential value in precisely activating anti-tumor immunity and enabling personalized treatment has been widely recognized (15). Unlike traditional vaccines, mRNA vaccines induce the production of specific tumor-associated antigens within the body by directly encoding mRNA sequences for these antigens. This effectively activates the immune system, particularly the cytotoxic T cell response dominated by CD8^+^ T cells, enabling the specific recognition and elimination of tumor cells. This immunological characteristic endows tumor mRNA vaccines with unparalleled advantages over traditional vaccines in terms of antigen design flexibility, target renewal speed, combinatorial immunotherapy adaptability, and personalized customization (16). However, naked mRNA is highly susceptible to degradation and exhibits low cellular uptake efficiency, which substantially limits its clinical application. Among available approaches, micelle-based vaccine formulations offer advantages such as efficient cytoplasmic antigen delivery and induction of dendritic cell (DC) activation. mRNA lipid nanoparticles incorporating cross-linked ionizable lipids can not only modulate the functional state and metabolic processes of immune cells, but also efficiently elicit vaccine-induced immune responses while markedly reducing pro-inflammatory side effects. Collectively, advances in these delivery technologies have driven the development of promising therapeutic cancer vaccine platforms and laid a solid foundation for their clinical translation (17, 18).

The tumor microenvironment (TME) plays a crucial role in determining the extent and persistence of tumor immune therapy responses (19). In solid tumors such as lung cancer, the TME often exhibits pronounced immunosuppressive characteristics, including abnormal accumulation of regulatory immune cells (such as regulatory T cells (Tregs), myeloid-derived suppressor cells (MDSCs), and tumor-associated macrophages), massive secretion of inhibitory cytokines like TGF-β and IL-10, and polarization of tumor-associated macrophages toward an immunosuppressive phenotype. Among these, TAMs profoundly influence tumorigenesis, progression, and immune regulation by participating in multiple processes, including the modulation of antigen presentation, promotion of angiogenesis, and remodeling of the extracellular matrix. In particular, M2-like TAMs have emerged as a major focus of cancer research (20). These factors collectively impair effector T cell function, enabling tumor cells to evade immune surveillance and continue growing and metastasizing (21). Therefore, targeted interventions targeting the tumor microenvironment have emerged as a research hotspot in tumor immunology in recent years, with mRNA-based therapeutic vaccines regarded as a key potential strategy for reshaping the TME. The TME remodeling it mediates encompasses dual mechanisms: direct local initiation driven by the vector or encoded factors, and downstream activation of antigen-specific T cells. Existing preclinical and early clinical studies indicate that mRNA vaccines not only effectively avoid common adverse reactions during treatment but also demonstrate reliable safety and tolerability (22, 23). Moreover, mRNA vaccines can simultaneously encode multiple tumor-associated antigens, offering the potential for personalized therapies targeting mutations such as *KRAS*. They can also amplify and sustain antitumor immune responses by promoting dendritic cell maturation and reprogramming macrophages (24–26). Additionally, mRNA vaccines can be combined with other immunotherapy such as ICIs to synergistically improve tumor microenvironment, partially overcoming tumor immune escape and thereby enhancing treatment efficacy (27). Although the application of therapeutic mRNA vaccines for cancer remains in the early stages of research and clinical translation, their unique advantages in precisely regulating anti-tumor immune responses, enabling personalized treatment, and reshaping the tumor microenvironment offer new approaches and methodologies for future cancer immunotherapy.

Building on the above background, this review approaches the topic from the perspective of mRNA vaccines as reprogrammers of the tumor microenvironment, and systematically examines their functional evolution from the passive recognition of tumor antigens to the active remodeling of the immune microenvironment. The article is organized around the following logical framework: identification of reprogramming targets, elucidation of reprogramming mechanisms, validation of reprogramming efficacy, and realization of precise reprogramming, with the aim of establishing a more comprehensive theoretical foundation for the clinical translation of mRNA vaccines.

## 2 Comprehensive identification of potential tumor antigens and ferroptosis-related immune subtypes in lung cancer along with corresponding immune biomarkers

**Figure 1:**
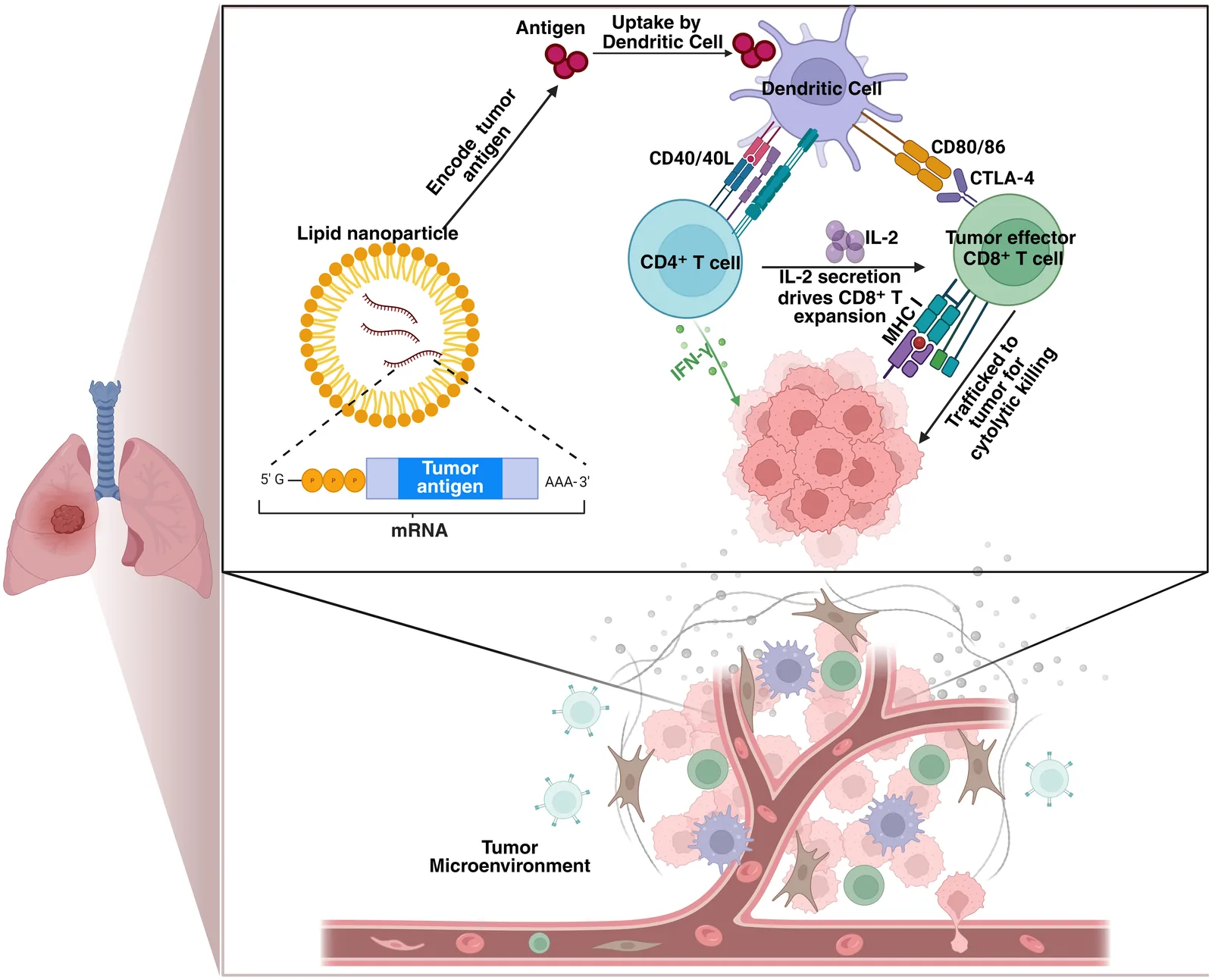
The cellular immune mechanism induced by mRNA vaccines against tumor cells in the lung cancer tumor microenvironment. After mRNA encoding lung tumor antigens is encapsulated by LNP, it is taken up by dendritic cells (DCs). The tumor antigens translated within the cells undergo processing and are presented on major histocompatibility complex (MHC) molecules. Antigen-presenting DCs activate naive CD4^+^ T cells through CD40/CD40L and other co-stimulatory signals. Activated CD4^+^ T cells secrete IL-2, thereby driving the clonal expansion and effector differentiation of tumor-specific CD8^+^ T cells. Simultaneously, signals mediated by co-stimulatory/co-inhibitory molecules such as CD80/86 and CTLA-4 between DCs and CD8^+^ T cells further regulate the activation state of CD8^+^ T cells. Based on this, tumor-specific effector CD8^+^ T cells generated through differentiation subsequently migrate to the lung tumor site, recognize tumor antigens, and mediate cytotoxic killing of malignant cells—a process partially dependent on cytokines such as IFN-γ.

The immunogenicity, exposure site, and toxicity of antigens collectively determine the efficacy of the immune response (28–30). Therefore, selecting antigens with high immunogenicity, low toxicity, and the ability to expose dominant sites within the immune system is key to enhancing the level of immune response (31). This choice is crucial for the effectiveness of lung cancer immunotherapy strategies. As shown in **Figure 1**, mRNA vaccines generally enhance the immune efficacy of tumor cell killing by promoting the recruitment and activation of immune cells through their unique cellular immune mechanisms. Through this process, mRNA vaccines can effectively guide the immune system to recognize and attack tumor antigens associated with lung cancer. These classic tumor-associated antigens serve not only as important diagnostic markers for lung cancer but also as key targets for developing tumor vaccines (**Table 1**).

**Table 1:** Recent advances in research on classic tumor antigens in lung cancer.

Antigen name	Antigen category	Frequency of expression	Immunogenicity	Research applications	References
EGFR	Epidermal Growth Factor Receptor	/	High (Enhances immune cells’ recognition of tumor cells and cytotoxic effects)	Tyrosine kinase inhibitor	(42)
HER2	Oncogene/Target protein	7.7%–23.0% (overexpression)	HER2 amplification correlates with PD-L1 expression, demonstrating strong immunogenicity in NSCLC.	Novel Antibody-Drug Conjugates and Tyrosine Kinase Inhibitors	(43, 44)
*TP53*	Tumor Suppressor Gene/Transcription Factor	33%–90% (mutation)	/	Restoring p53 Function Using APR-246 and HSP90 Inhibitors; Treating GOF p53 with Proteasome Inhibitors	(45)
MUC1	Mucin 1	Abnormally overexpressed in over 80% of stage IB NSCLC cases	High (potential immunogenicity)	Investigating MUC1-C as a Target for Downregulating PD-L1	(46)
WT1	tumor suppressor gene	/	/	The interaction between WT1 and IL-10 may serve as a potential therapeutic target for modulating the immune response in lung cancer.	(47)
MAGE-A3	Cancer—Testicular Antigen	Highly expressed in NSCLC	Activate CD8^+^ T cells, particularly those recognizing the epitope Mp4.	TCR-T Immunotherapy Targets	(48)
MAGE-A4	Cancer—Testicular Antigen	Expressed in 22%–38% of NSCLC cases	Promote plasma cell accumulation and suppress T-cell response	Immunotherapy Targets, Modulating the Immune Microenvironment	(49)

In addition, this paper also provides an objective assessment of several potential novel tumor antigen targets with promising application prospects. The L523S mutation, a marker for carcinoembryonic antigen, exhibits a positive expression rate of up to approximately 80% in non-small cell lung cancer tissues, whereas its expression in normal tissues is relatively limited (32), Predina et al. have engineered L523S into an adenovirus-based tumor vaccine and demonstrated its favorable safety profile in clinical trials, with only minimal adverse effects observed (33). mRNA level detection combined with immunohistochemistry results revealed that L523S is significantly expressed in 80-90% of NSCLC specimens, making it a potentially valuable therapeutic target with broad applicability across the NSCLC population (34, 35). Immunotherapy strategies based on L523S demonstrate considerable potential for clinical translation, particularly in activating and amplifying specific antitumor immune responses. Xu et al. performed KEGG enrichment analysis on copy number variation genes in NSCLC patients and combined them with mutated genes from The Cancer Genome Atlas database. They identified 13 potential LUAD antigens, including *TTK*, *BUB1B*, *ASB2*, *HJURP*, *CBFA2T3*, *PTGFRN*, *HSPA4*, *ST6GAL1*, *CENPU*, *KLRG1*, *GTF3C6*, *OIP5*, and *VDAC3*. The elevated expression and prognostic associations of these genes support their prioritization as candidate antigen sources for further investigation. In particular, *KLRG1* and *CBFA2T3* may represent potentially relevant candidates in early-stage LUAD. However, transcript overexpression and bioinformatic association alone do not demonstrate that peptides derived from these proteins are naturally processed and presented by patient HLA molecules or recognized by tumor-reactive T cells. Therefore, direct validation of peptide-HLA presentation and antigen-specific T-cell immunogenicity is required before these candidates can be regarded as vaccine-ready targets (36). Wei et al. employed dual-cohort transcriptomic differential analysis (GSE60052, GSE30219; log_2_|FC| ≥3, FDR < 0.05) and intersected these results with the somatic mutation gene set (n=11093) from cBioPortal, yielding nine candidate genes. In small cell lung cancer (SCLC) cell lines (H446, H1688) and normal lung epithelial cells (BEAS-2B), PCR analysis of mRNA levels revealed that only *NEK2*, *NOL4*, *RALYL*, *SH3GL2*, and *ZIC2* were significantly overexpressed in SCLC cell lines. Consequently, *NEK2*, *NOL4*, *RALYL*, *SH3GL2*, and *ZIC2* were identified as potential mRNA vaccine antigen candidates for SCLC. Their relatively high expression in the Immunity_L subtype suggests potential value for patient stratification and personalized immunotherapy. Nevertheless, their HLA presentation and ability to induce functional antigen-specific T-cell responses require further validation (37).

In the screening of immune biomarkers, Wang et al. identified a set of potential novel LUAD immune-related markers through bioinformatics analysis, including *GPRIN1*, *MYRF*, *PLXNB2*, *SLC9A4*, *TRIM29*, *UBA6*, and *XDH*. These immune markers not only provide new targets for targeted immunotherapy but also hold promise as important biomarkers in precision immunotherapy. Wang et al. further revealed the immune subtype (Immune Subtypes1-5; IS1-IS5) clustering patterns in LUAD, with IS2 and IS4 patients exhibiting higher tumor mutational burden and a richer somatic mutation spectrum. This suggests that this population is more likely to benefit from personalized immunotherapy regimens targeting patients with high TMB (38). Furthermore, the role of ferroptosis in tumor immunotherapy and its potential application value are also gaining increasing attention (39, 40). Through analysis of iron apoptosis subtypes (Ferroptosis Subtypes 1-3; FS1-FS3), Chen et al. found that FS3 exhibited the highest tumor mutation burden and the greatest number of somatic mutations, making it a recommended potential indicator of vaccine efficacy. In FS3 subtype patients, the *AGPS*, *NRAS*, *MTDH*, *PANX1*, *NOX4*, and *PPARD* genes show promise as effective vaccination targets, providing crucial evidence for future immunotherapy strategies (41).

Existing research has identified multiple immunologically relevant biomarkers with translational potential for lung cancer immunotherapy. Among these, antigen targets such as L523S, *KLRG1*, and *CBFA2T3* demonstrate strong potential. Notably, L523S exhibits widespread and relatively specific expression patterns in NSCLC, making it a significant candidate target for immunotherapy. In early-stage patients, the high expression of *KLRG1* and *CBFA2T3* suggests that these antigens hold promise as important targets for enhancing the efficacy of immunotherapy. Meanwhile, the subtype analysis based on iron apoptosis characteristics offers a novel stratification approach for personalized immunotherapy. Among these, patients with the FS3 subtype exhibit tumor mutation burden and somatic mutation profiles that make them a key target group for immunotherapy. While these immune biomarkers demonstrate promising clinical potential, further clinical data is still required to validate their actual immunological efficacy and safety. Overall, these studies have not only deepened our understanding of the immune heterogeneity of lung cancer, but also provided important clues for the selection of antigenic targets for mRNA vaccines. Accordingly, the identification of antigenic targets has laid the foundation for the therapeutic application of mRNA vaccines. However, merely defining the relevant targets is insufficient to fully account for their therapeutic potential. More importantly, the key question is how mRNA vaccines can exploit these targets to achieve systematic modulation of the tumor microenvironment. With this central issue in mind, the following section further discusses the mechanisms by which mRNA vaccines mediate remodeling of the tumor microenvironment.

## 3 mRNA vaccines precisely modulate the lung cancer tumor microenvironment

Precise modulation of the tumor microenvironment is the key to enabling therapeutic tumor vaccines to play a dominant role (50). mRNA vaccines leverage their unique advantages to not only promote the generation and functional regulation of both immune-activating and immune-suppressive cells, but also reshape the spatial distribution of immune cells within the tumor microenvironment. They effectively induce the transformation of tumors from “cold tumors” to “hot tumors” (51). These mechanisms play an indispensable key role in regulating the tumor immune microenvironment. mRNA vaccines are linear single-stranded RNA molecules composed of an open reading frame, a 5’ cap structure, untranslated regions, and a 3’ polyadenylate tail. Due to the strong negative charge of free mRNA and its susceptibility to degradation by nucleases, its application *in vivo* necessitates reliance on suitable delivery vehicles for protection and efficient transport. Currently, the most commonly reported protective carriers primarily include liposomes (52–54) and lipid nanoparticles (55–57).

mRNA vaccines have emerged as a promising approach to modulate the lung cancer tumor microenvironment, offering potential therapeutic benefits by targeting immune components within this complex setting, with a focus on redirecting them toward antitumor activity (58, 59). The tumor microenvironment is a key determinant in the development and progression of primary lung cancer, exhibiting high heterogeneity. This heterogeneity complicates the mutations in cancer cells while simultaneously driving tumors toward a direction that is difficult to control, posing greater challenges for treatment. Within the lung cancer microenvironment, malignant tumor cells can reprogram infiltrating stromal cells and immune cells through the secretion of multiple soluble factors and cell-contact-dependent signaling. This leads to the functional exhaustion or inactivation of cytotoxic CD8^+^ T cells, which should otherwise play a critical anti-tumor role (60, 61). The tumor immune microenvironment (TIME) plays a decisive role in the initiation, progression, invasion, and distant metastasis of lung cancer. The dynamic interactions among its diverse cellular components and structural elements collectively shape characteristics such as the invasive and metastatic behavior of lung cancer cells, fundamentally reflecting an imbalance between immune suppression and cytotoxic responses (62, 63). The tumor microenvironment of lung cancer readily forms a highly complex immunosuppressive network, in which cell populations such as Tregs, MDSCs, and tumor-associated macrophages exhibiting immunosuppressive phenotypes often dominate. These cells significantly suppress immune activity by secreting immunosuppressive factors such as TGF-β and IL-10, or by directly interacting with effector T cells like CTLs, thereby further diminishing the efficacy of antitumor immune responses (64). Against this backdrop, the sustained overexpression of immunosuppressive factors disrupts the dynamic equilibrium between immunosuppressive and immunostimulatory signals, thereby limiting the direct cytotoxic effects of CTLs on tumor cells (65). mRNA vaccines can effectively utilize the body’s protein synthesis system to encode multiple tumor-associated antigens (TAAs). These antigens are presented to CD8^+^ T lymphocytes by binding to the major histocompatibility complex (MHC-I) on antigen-presenting cells (APCs). Driven by the APC’s MYD88 or TRIF signaling pathways, this process activates the transcription factor NF-κB, inducing the transcription and expression of immune-related genes such as multiple cytokines and co-stimulatory molecules. This effectively promotes the proliferation and effector functions of antigen-specific cytotoxic T lymphocytes, thereby inducing potent cytotoxic immune responses (66–68). Such immunotherapy strategies hold promise for suppressing or even reversing the progression of tumor metastasis to a certain extent (68).

Importantly, the reshaping of the immunosuppressive TME in this system operates through a coordinated mechanism: co-delivered immunostimulatory factors or vector adjuvants (such as IL-12 mRNA or mPLA) directly prime the local immune milieu to convert ‘cold’ tumors into ‘hot’ ones, whereas the resulting antigen-specific effector T cells act as the core secondary driver for sustained downstream TME reprogramming. IL-12 mRNA is considered a therapeutic tumor vaccine candidate with a solid biological rationale (69, 70). It achieves precise targeted delivery to lung cancer tumor cells via inhalable extracellular vesicle carriers, significantly increasing the concentration of cytotoxic CD8^+^ T cells in the local environment while effectively promoting the substantial recruitment of CD4^+^ T cells, natural killer (NK) cells, and monocytes. Transforming lung cancer from a “cold tumor” to a “hot tumor” creates the necessary conditions for effective infiltration by anti-tumor lymphocytes. With the assistance of extracellular vesicles in delivery, IL-12 mRNA provides critical signaling to anti-tumor lymphocytes (T cells and NK cells), thereby promoting the expansion of immune cell populations. This effectively reduces the accumulation and immunosuppressive functions of regulatory T cells and myeloid-derived suppressor cells, progressively breaking the immunosuppressive effects within the tumor microenvironment. Furthermore, IL-12 mRNA significantly enhances the expression of broad-spectrum inflammatory cytokines and chemokines within the TME, particularly through the marked upregulation of key chemokines CXCL9 and CXCL10. This plays a crucial role in transforming the TME into a “hot tumor” (71).

mRNA stimulates cancer antigen presentation by encoding specific cancer antigen epitopes, thereby effectively mitigating the suppression of CTL proliferation and activation caused by DC dysfunction. This mechanism significantly alleviates the adverse effects of the immunosuppressive network within the TME (72). Mature DCs further stimulate CD8^+^ T cell proliferation and induce tumor cell killing by releasing perforin to bind with FasL (73). From another perspective, macrophages in the TME are primarily categorized into classically activated M1 macrophages and alternatively activated M2 macrophages. These two macrophage types exert pro-inflammatory and immunosuppressive effects, respectively, in tumorigenesis and progression (74–76). The mPLA/mRNA lung cancer vaccine reprograms M2 macrophages into M1 macrophages, converting pro-tumor M2 macrophages (secreting anti-inflammatory factors such as IL-10 and TGF-β) into anti-tumor M1 macrophages (secreting pro-inflammatory factors such as IL-12 and TNF-α). NK cells also play an active role in the fight against tumors, serving as the host’s first line of defense against cancer. However, the interaction between NK cell immunoglobulin-like receptors and MHC-I ligands expressed on tumor cells generates inhibitory signals that lead to NK cell inactivation. Consequently, the activity and number of NK cells significantly influence the efficacy of antitumor effects (77). To enhance this effect and ensure synergistic antitumor responses within the microenvironment, stimulation with NK cell-specific ligands such as MICA/MICB, ULBPs, B7-H6, and antibody fragment crystallizable (Fc) fragments induces IFN-γ and IL-12 production, further promoting NK cell proliferation and amplifying antibody-dependent cytotoxicity mediated by natural killer cells (25). Through multiple immune regulatory mechanisms, mRNA vaccines can effectively remodel the tumor microenvironment by enhancing CTL responses and increasing the secretion of cytokines such as IL-12 and IFN-γ, while simultaneously reducing the proportion of M2 macrophages. This improves the immune properties of the tumor microenvironment and enhances its supportive role in immune responses (68).

**Figure 2:**
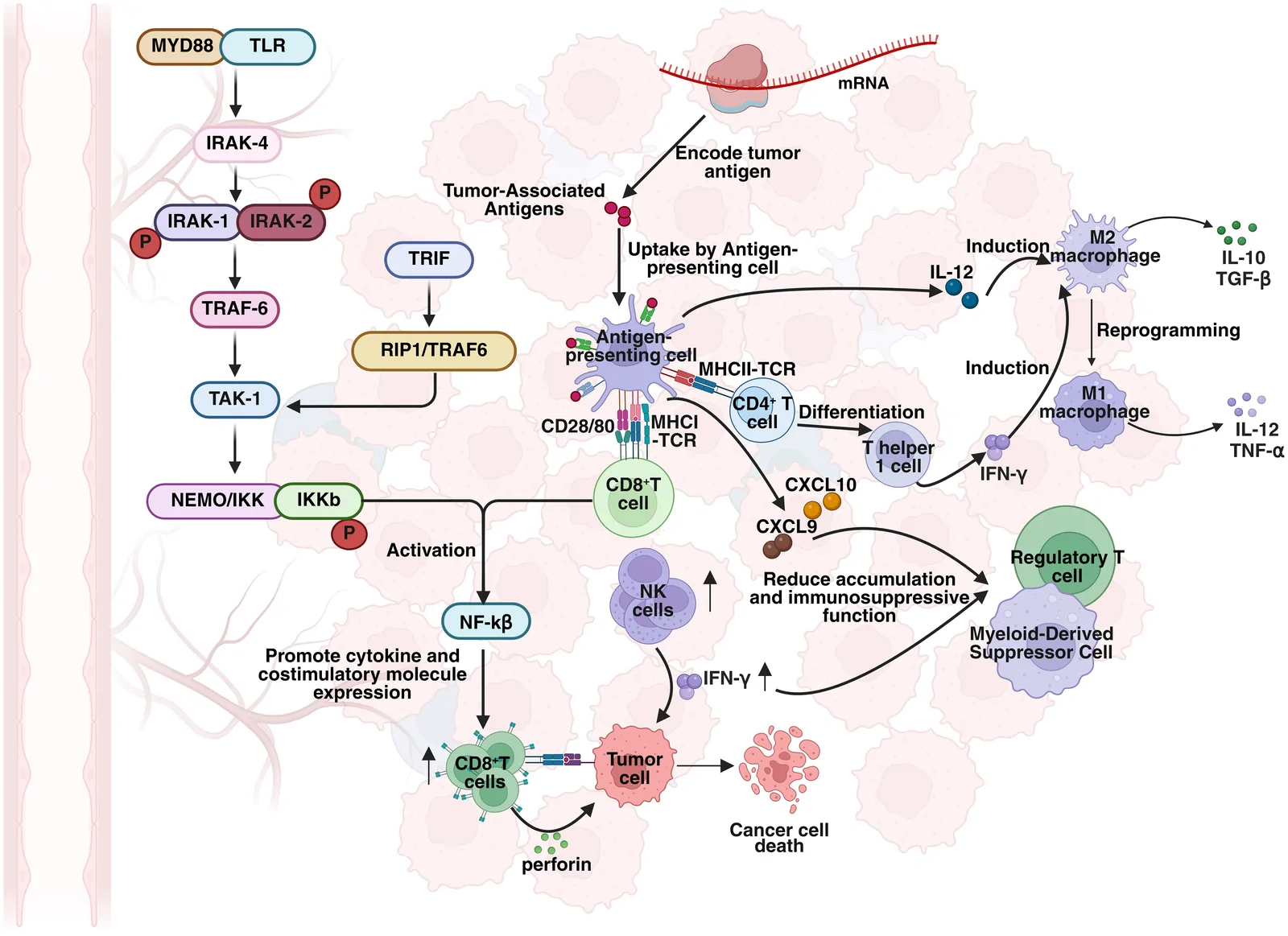
Schematic diagram of the mechanism by which mRNA vaccines remodel the lung cancer tumor microenvironment. After being delivered to the tumor microenvironment of lung cancer via lipid nanoparticles, mRNA vaccines encoding tumor-associated antigens are taken up by APCs. Within the cytoplasm, the mRNA is translated and expresses the corresponding tumor-associated antigen proteins. Antigens processed and presented via the MHC I and MHC II pathways, in combination with CD28/CD80 co-stimulatory signals, activate CD8^+^ T cells and CD4^+^ T cells. Upon activation of APC surface receptors, a MYD88 and TRIF dependent signaling cascade (IRAK1/2–TRAF6–TAK1–NEMO/IKKb) is initiated, leading to NF-κB activation and upregulation of multiple cytokines and co-stimulatory molecules. This promotes CD8^+^ T cell activation, proliferation, and enhances their cytotoxic effects against tumor cells. Simultaneously, the MHCII-TCR pathway promotes the differentiation of CD4^+^ T cells toward the Th1 phenotype. Th1 cells secrete IFN-γ to activate and expand NK cells while enhancing the effector function of CD8^+^ T cells, enabling activated CD8^+^ T cells to induce tumor cell lysis through perforin-mediated pathways. Concurrently, IL-12 secreted by APCs, along with IFN-γ produced by Th1 cells and NK cells, jointly drive the reprogramming of M2 macrophages into M1 macrophages. In this process, M1 macrophages secrete IL-12 and TNF-α, while M2 macrophages secrete IL-10 and TGF-β. Additionally, the secretion of CXCL9 and CXCL10 by APCs, along with IFN-γ produced by Th1 cells and NK cells, promotes the reduction of regulatory T cells and myeloid-derived suppressor cells, as well as their immunosuppressive activity.

Tregs are key immunosuppressive cells in the TIME. In various solid tumors, including lung cancer, Treg infiltration within tumor tissues is significantly elevated. The inhibitory cytokines they secrete, such as IL-10 and TGF-β, markedly attenuate the effector functions of CD4^+^ and CD8^+^ T cells. This reduces cytotoxic killing of tumor cells while simultaneously driving the formation and maintenance of an immunosuppressive microenvironment (78). To overcome this immunosuppressive barrier, mRNA vaccines can reshape the infiltration patterns of tumor-associated immune cells. By promoting the recruitment of antitumor immune cells such as effector T cells and dendritic cells while suppressing the accumulation of tumor-associated regulatory T cells and myeloid suppressor cells, they demonstrate potential for modulating immune responses in cancer therapy (79). For example, flow cytometric analysis of mPTEN(phosphatase and tensin homolog deleted on chromosome 10 messenger RNA)@NPs developed by Lin et al. confirmed a significant reduction in the numbers of Tregs and monocytic MDSCs (Mo-MDSCs). Mechanistic studies indicated that this change did not result from direct cytotoxicity of mPTEN@NPs against Tregs or MDSCs. Instead, restoration of PTEN expression induced tumor cell death and the subsequent release of damage-associated molecular patterns, which in turn promoted the secretion of pro-inflammatory cytokines such as IL-12, TNF-α, and IFN-γ, thereby indirectly suppressing the local accumulation of Tregs and Mo-MDSCs within the tumor (80). Following vaccination with the MoVA/bIL15-LNP vaccine constructed by Zhang et al., the proportion of immunosuppressive Tregs showed a sustained decline. This effect is speculated to result from the vaccine’s ability to block both exogenous recruitment of Tregs by the tumor and their local endogenous induction (81). This shift in cellular infiltration patterns is primarily achieved by regulating the expression of key chemokines and cytokines (such as CXCL9, CXCL10, and IL-12), which are crucial for the migration and activation of immune cells (82). Specifically, research indicates that mRNA vaccines based on nanostructured lipid carriers can significantly upregulate these CD8^+^ T cell recruiting chemokines. This promotes the infiltration of effector T cells into the tumor microenvironment and reshapes the composition of immunosuppressive myeloid cells (including M2-like tumor-associated macrophages). Consequently, “cold” tumors are transformed into T-cell inflammatory microenvironments, further enhancing the antitumor immune response (27) (**Figure 2**).

Moreover, the anti-tumor immunity elicited by mRNA vaccines is not solely driven by a single antigen. Rather, it results from the synergistic remodeling of the TME by functional components, including the mRNA construct, its delivery system, and adjuvants. The rational design of these components holds immense promise for targeting and reprogramming specific immunosuppressive cell populations, such as TAMs and Tregs. For instance, LNP-mediated co-delivery of mutant KRAS G12D mRNA and the STING agonist cGAMP induces type I interferon signaling, successfully reprogramming immunosuppressive cells into an immunostimulatory phenotype and thereby thoroughly dismantling the immunoprotective niche of pancreatic cancer liver metastases (83). For TAMs, incorporating specific Toll-like receptor (TLR) agonists into the vaccine formulation can directly reverse their polarization state. For instance, integrating the TLR4 agonist mPLA into an mRNA lipoplex vaccine precisely activates the TLR4/JAK2/STAT1 signaling pathway, driving the repolarization of pro-tumorigenic M2 TAMs toward the highly anti-tumor M1 phenotype. This process not only substantially reduces the M2/M1 ratio but also synergistically promotes the deep infiltration of CD8^+^ T cells (25). For Tregs, emerging carrier engineering technologies offer a similarly effective approach. For instance, conjugating IL-2 to the surface of LNPs leverages the constitutively high expression of the IL-2 receptor (CD25) on Tregs to deliver mRNA precisely and specifically into FoxP3^+^ Tregs. This direct, active targeting strategy serves as a compelling paradigm for mRNA vaccines to reverse TME immunosuppression by modulating specific immunosuppressive subsets (84). However, due to the profound heterogeneity of MDSCs and the absence of specific surface markers, direct and precise active targeting of these cells by mRNA vaccines remains challenging. Consequently, relevant studies are scarce at present.

Different lung cancer immune subtypes exert a significant influence on the composition and distribution of the tumor microenvironment (85–87). Analysis by Zhao et al. using the Immune Risk Score (IRS) scoring system at the single-cell level revealed that in the microenvironment of immune-resistant subtypes of lung adenocarcinoma, the proportion of tumor cells was significantly elevated. In contrast, the immune-active subtype exhibited a higher proportion of anti-tumor immune cells. It is speculated that this may be due to the activation of the CXCL-CXCR pathway in the immune-active subtype. This pathway can recruit more T cells or B cells into the tumor microenvironment, thereby enhancing interactions among immune cells. These characteristics make immune-active subtype tumors a classic “hot” tumor type, exhibiting strong immune responses and immune cell infiltration capacity, and thus more likely to respond well to immunotherapy (88, 89). Therefore, the antigenic proteins produced by mRNA vaccines through translation activate specific antitumor immune responses. This approach holds promise for reshaping the tumor immune microenvironment of “cold” subtype lung adenocarcinoma to some extent, promoting its transition toward the “hot” subtype. Consequently, it enhances the response rate to immunotherapy and establishes a robust immunological foundation for subsequent targeted treatments. In summary, the mechanistic studies discussed above have elucidated the molecular basis by which mRNA vaccines remodel the tumor microenvironment. However, whether these theoretical advantages can be translated into tangible clinical benefits for patients in real-world practice still requires validation through clinical trials.

## 4 Research progress and clinical translation of lung cancer mRNA vaccines

CTLA-4 and PD-1 are two key inhibitory immune checkpoint pathways regulating T cell responses. They negatively modulate T cell activation during the sensitization and effector phases, thereby limiting their antitumor effector functions (90). Immune checkpoint inhibitors targeting the PD-1/PD-L1 axis block the interaction between PD-1 and PD-L1, thereby releasing the functional suppression of effector T cells and restoring and enhancing the body’s immune response against tumors (91). Therefore, PD-1/PD-L1 monotherapy is recommended as the standard treatment for patients with non-squamous (NS)-NSCLC (92). In contrast, CTLA-4 inhibitors primarily exert their effects by blocking the negative regulation of CD28-B7 co-stimulation mediated by CTLA-4 during sensitization. They selectively reduce intratumoral Tregs, thereby increasing the Teff/Treg ratio. This mechanism generates complementary and synergistic antitumor effects when combined with PD-1/PD-L1 inhibitors (93). Although immune checkpoint inhibitors have demonstrated some efficacy in lung cancer treatment, the overall response rate remains low, resulting in limited therapeutic benefits (5). In contrast, mRNA vaccines, by encoding and synthesizing tumor-specific antigens (TSAs) or tumor-associated antigens, present them through MHC class I and MHC class II molecular pathways, respectively. This approach simultaneously activates cytotoxic CD8^+^ T cell-mediated targeted killing and CD4^+^ T cell-dependent cytokine network regulation, thereby reshaping and amplifying the anti-tumor immune response. This holds promise for the combined application of mRNA vaccines with immune checkpoint inhibitors (94–96). To effectively translate this immunological advantage into a mucosal delivery strategy for lung tumors, Liu et al. developed a charge-assisted stabilization (CAS) strategy using peptide-lipid conjugates to optimize surface charge. This approach addressed the issue of LNP degradation and aggregation during nebulization for lung cancer mucosal mRNA vaccines, enhancing electrostatic repulsion to increase stability. The experiments used CAS-LNP and SM102-LNP to encapsulate mRNA encoding ovalbumin (mOVA). The results demonstrated that compared to the SM102-LNP-mRNA group, CAS-LNP-mRNA increased the percentage of OVA-specific IFN-γ^+^CD8^+^ T cells by approximately 8.7-fold and 21.4-fold, respectively. This triggered a systemic antitumor immune response, thereby achieving the therapeutic effect of inhibiting lung metastasis. To further investigate the efficacy of the CAS-LNP-mRNA vaccine, this study developed an mRNA vaccine encoding the natural B16F10 tumor antigen, the 70-kDa envelope glycoprotein, and validated its efficacy in a mouse lung metastasis model. The results demonstrate that this vaccine significantly reduces the number of pulmonary metastatic lesions and optimizes the M1/M2 ratio, showing promising potential for application in lung cancer treatment (97). mRNA vaccines engineered for multiple *KRAS* mutations have demonstrated promising antitumor effects in preclinical studies (22, 98, 99). In the LL/2 homozygous mouse cancer model (Lewis lung carcinoma, *KRAS*^G12C^ mutant), the *KRAS* mRNA vaccine demonstrated a significant inhibitory effect on the growth of LL/2 tumors within an immunosuppressive tumor microenvironment (22).

Based on the aforementioned preclinical evidence, mRNA vaccines for lung cancer have demonstrated biological feasibility for human studies. Concurrently, given the genetic heterogeneity and immunosuppressive microenvironment of NSCLC, the current clinical translation phase primarily pursues two pathways: universal multi-antigen combinations and personalized neoantigens. These approaches have already provided preliminary evidence of the efficacy and safety of these lung cancer mRNA vaccines in human populations (87). **Table 2** systematically compiles currently registered or ongoing clinical studies of mRNA vaccines for lung cancer, providing a clear overview of key vaccine information (**Table 2**). However, no mRNA lung cancer vaccine has yet been approved for clinical use. Among them, the CV9201- and CV9202-related mRNA vaccines induced peripheral blood-specific immune responses in as many as 63%-84% of cases, yet the ORR approached zero, revealing a marked disconnect between immunological readouts and clinical benefit. This discrepancy is presumed to stem from dual constraints at both the biological and technical-delivery levels. Biologically, constrained by host central immune tolerance, TAA-induced T cells generally exhibit low affinity for tumor antigens and limited cytotoxic efficacy (100). Meanwhile, the substantial tumor burden in patients with advanced NSCLC markedly impairs vaccine-induced immune clearance. Even when combined with local radiotherapy, this limitation remains difficult to overcome (101). More critically, the highly immunosuppressive TME of lung cancer drives rapid exhaustion of infiltrating T cells through persistent antigen exposure, metabolic suppressors such as adenosine and cholesterol, and upregulation of inhibitory receptors including PD-1 and lymphocyte activation gene 3(LAG-3) (102). Secondly, at the level of delivery technology, the low delivery efficiency and insufficient cellular uptake associated with syringe-injected vaccines further constrain antitumor efficacy (103). To overcome the dual barriers of immune tolerance and delivery, current research should focus on developing efficient LNP delivery systems, optimizing administration routes, and establishing personalized neoantigen-targeted platforms, thereby enabling durable systemic antitumor immune responses (104, 105).

**Table 2:** Clinical development progress of select mRNA therapeutic vaccines for lung cancer.

Vaccine name	Target antigen	Combination drugs	Antigen strategy	Immunogenicity	Clinical efficacy	Research and development phase	Safety	Source
BNT116	CLDN6, KK-LC-1 MAGE-A3 MAGE-A4 MAGE-C1 PRAME	Cemiplimab	Encoding multiple tumor-associated antigens commonly expressed in NSCLC	The patient demonstrated a robust type I interferon response and a multifaceted T-cell response following treatment	ORR: 45% Disease Control Rate(DCR): 80% Median Progression-Free Survival(PFS)9.9 months	Phase I/IIa	Controllable safety profile: 15% of patients experienced ≥G3 BNT116-related adverse reactions, while 65% experienced Cemiplimab-related adverse reactions. No fatal adverse reactions were reported	(106)
BMD006	Six Primary Lung Cancer-Associated Antigens	PD-1 antibody	Encoding multiple tumor-associated antigens	/	/	Early Phase 1	/	(107)
ABO2013	EGFR mutation	Sintilimab	EGFR mutation-encoded antigen	Significant neoantigen-specific immune responses were observed	Some patients achieved complete or partial remission	Phase I	No dose-limiting toxicity or Grade 3–4 adverse events were observed	(108, 109)
CV9201	NY-ESO-1, MAGE-C1, MAGE-C2, Survivin, 5T4	/	Encoding five antigens commonly expressed in NSCLC	63% of evaluable patients developed antigen-specific immune responses, and in 60% of patients, the frequency of activated IgD^+^CD38^hi B cells increased more than twofold	Median PFS was 5 months; median overall survival was 10.8 months;no objective tumor responses were observed	Phase I/IIa	CV9201 was well tolerated, with common adverse reactions including mild to moderate injection site reactions and flu-like symptoms. Grade 3 adverse reactions occurred in 7% of patients	(105)
CV9202	NY-ESO-1 MAGE-C1 MAGE-C2 Survivin 5T4 MUC-1	/	An adjuvant and six mRNA sequences encoding tumor-associated antigens	84% of patients developed enhanced antigen-specific immune responses following treatment	Approximately 46% of patients demonstrated stable disease, and one patient achieved partial remission; None of the patients who received only the vaccine and local radiotherapy achieved an objective response	Phase Ib	Safety profile was favorable, with adverse reactions primarily mild to moderate in severity. Four patients experienced Grade 3 adverse reactions, and there were no fatal events or immune-related adverse reactions	(104)
CVHNLC	Squamous NSCLC (specific antigen panel not disclosed/personalized)	pembrolizumab	Personalized/Precision mRNA Vaccines	Enhance tumor immune response	/	Phase I	/	(110)
mRNA-4157/V940	Individualized Neoantigens	pembrolizumab	Personalized neoantigen mRNA vaccine	T cell responses have been demonstrated in solid tumors (including NSCLC)	/	Phase III	/	(111)
mRNA-5671/V941	/	pembrolizumab	/	Mutant KRAS-specific T-cell responses were observed	ORR in the V941 + pembrolizumab group: 5.0%	Phase I	The rate of serious adverse events in the V941 + pembrolizumab group was 25%	(112)

## 5 LNP modification strategies in mRNA vaccine delivery systems and their auxiliary role in the tumor microenvironment

Because mRNA molecules are relatively unstable and prone to degradation within the body, delivery systems are required to protect their structure and facilitate their entry into cells. The delivery system for mRNA vaccines is a critical factor in ensuring their efficacy and safety, primarily encompassing polymer-based delivery systems (113), peptide-based delivery System (114), DC delivery (115), self-amplifying RNA (116). In particular, LNP is currently the most effective delivery system, validated through clinical trials of COVID-19 mRNA vaccines such as BNT162b2 and mRNA-1273 (117, 118). LNP is a nanoscale lipid carrier capable of efficiently encapsulating mRNA and delivering it into cells, while providing protection against mRNA degradation *in vivo*. When administered via intratumoral injection, it significantly reduces systemic toxicity. In particular, its multi-component design characteristics—including ionizable lipids, cholesterol, auxiliary lipids, and polyethylene glycol (PEG) lipids—are crucial for enhancing delivery efficiency and safety (116, 119–121). In particular, third-generation LNPs such as ALC-0315 and SM-102 have demonstrated highly efficient *in vivo* transfection of long-chain mRNA during experimental studies (16).

Research indicates that delivering mRNA to the TME using lipid nanoparticles is a highly viable approach capable of effectively activating immune protection *in vivo (*122). In recent years, research on modifying LNP delivery systems has proliferated, encompassing optimization of LNP composition, development of novel lipid reservoirs, and targeted modifications and functionalization. These efforts aim to enhance the immunostimulatory effects of LNP-mRNA in the tumor microenvironment. Optimizing the lipid composition and ratio of LNP systems has been demonstrated to be a critical factor determining their delivery efficiency, targeting specificity, and overall formulation stability (123). Kubara et al. employed LNPs formulated with the novel L17-F05 composition. By optimizing the composition of ionizable lipids, they achieved efficient delivery of mRNA to conventional dendritic cells (cDCs) and macrophages in draining lymph nodes. This significantly enhanced the efficiency of tumor antigen presentation. Furthermore, by activating the STING-IFN-I pathway, they induced an innate immune response, thereby further activating antitumor immune effects (124). Yang et al. developed a novel AOLs(amine N-oxide lipids)-LNP system. Through selective experiments, they found that compared to SM-102 LNP, the novel AOL-based LNP increased the number of migratory and resident cDC1 cells in muscle and lymph nodes, promoted intratumoral CD8^+^ T cell infiltration, and suppressed melanoma growth while reducing CD3^+^ CD45^+^ T cell infiltration in the liver. This approach thereby decreased the risk of hepatic inflammation (125). Liu et al. independently designed and synthesized a novel ionizable lipid library (DALs) featuring diamino headgroups. The preferred compound DAL4 was assembled with DOPE/cholesterol/DMG-PEG to form DAL4-LNP. Results demonstrated that this platform exhibited significantly superior *in vitro* delivery efficiency compared to MC3-type LNPs. Intratumoral injection of DAL4-LNP carrying IL-12 mRNA significantly suppressed B16F10 melanoma growth. When combined with IL-27 mRNA, it produced synergistic effects, inducing substantial NK cell and CD8^+^ T cell infiltration. This rapidly established a “hot tumor” microenvironment, leading to complete tumor regression in most mice and the establishment of long-term immune memory. Repeated administration showed no systemic inflammation or toxicity (122). Bevers et al. employed a quality design strategy based on experimental design and Bayesian regression modeling to optimize the LNP formulation (optimizing the molar ratio among the four lipids and selecting appropriate PEG lipids), thereby directionally enhancing tumor-specific CD8^+^ T cell responses (126). Additionally, Zhu et al. employed DLin-MC3-DMA as an ionizable lipid, DMG-PEG2000 as the PEGylated lipid, and six auxiliary phospholipids previously used in FDA-approved or experimental LNP formulations. They constructed an LNP library containing 1080 members and screened for the LNP formulation composition with the highest transfection efficiency and antigen presentation capacity by optimizing the type of auxiliary lipids and the lipid composition ratio. Subsequently, the top-performing LNP identified through high-throughput screening was administered to mice via a three-dose regimen, eliciting potent antigen-specific Th1 responses with significant tumor-suppressive effects (127). Therefore, LNP not only enhances antigen-specific immune responses through mRNA delivery but also reduces immunosuppressive cell infiltration while activating key immune cells. This provides a safe and efficient universal platform for tumor immunotherapy, particularly for lung cancer treatment.

**Figure 3:**
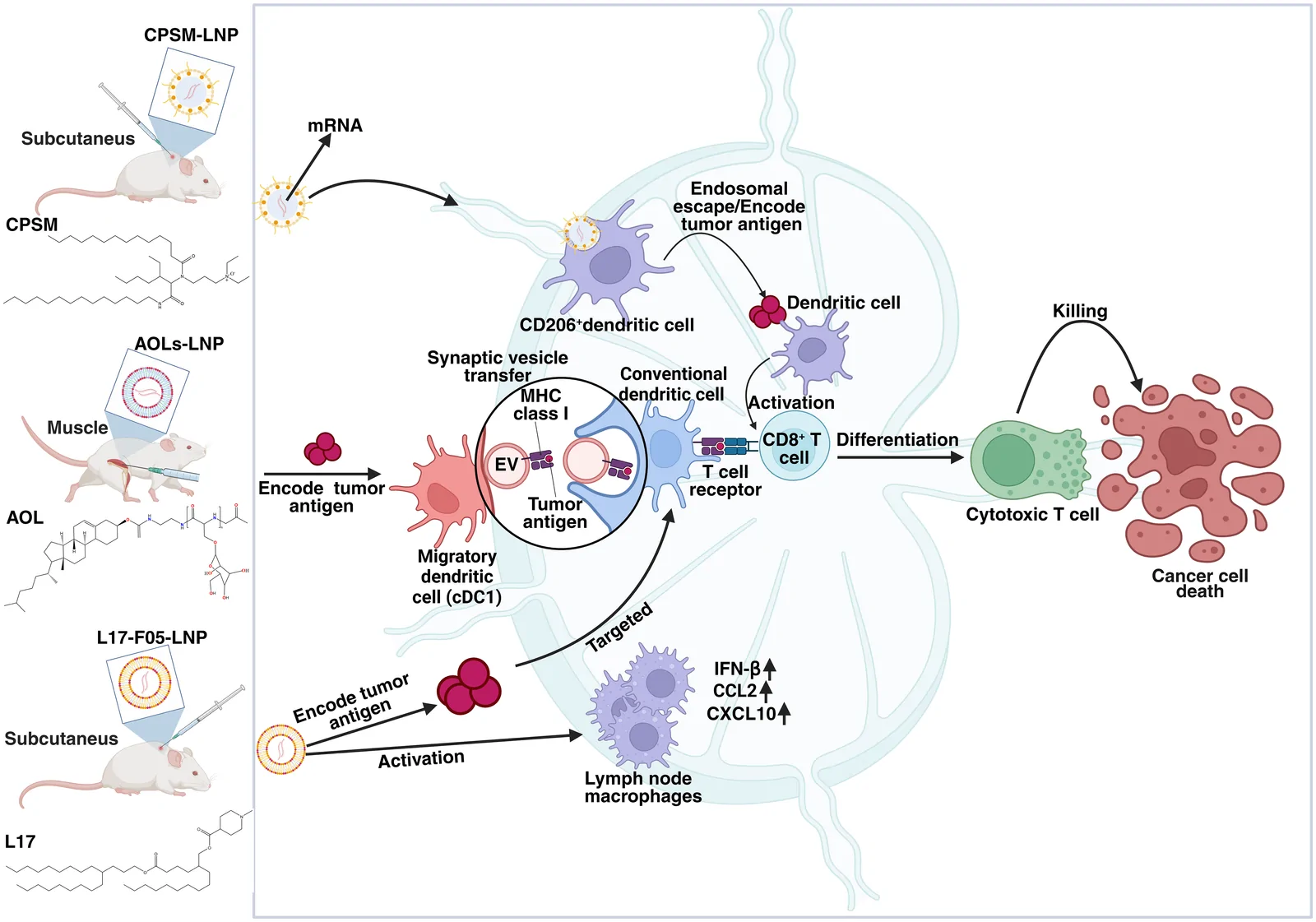
Schematic illustration of the mechanism by which three representative novel LNP-mRNA vaccines target antigen-presenting cells in tumor-draining lymph nodes and induce CD8^+^ T cell-mediated antitumor immunity. mRNA encapsulated in CPSM-LNP, AOLs-LNP, and L17-F05-LNP was administered subcutaneously or intramuscularly, subsequently draining via lymphatic vessels into the tumor-draining lymph nodes. Following subcutaneous injection of CPSM-LNP-mRNA, the complex is internalized by CD206^+^ dendritic cells within lymph nodes. After escaping the endosomal compartment, the mRNA encoding tumor antigens is released into the cytoplasm. The translated tumor antigens undergo processing and presentation, enabling activated dendritic cells to deliver both antigen and co-stimulatory signals to CD8^+^ T cells. This process drives the activation and differentiation of these cells into effector cytotoxic T cells. In the AOLs-LNP strategy, intramuscularly administered mRNA vaccines are efficiently taken up and expressed by antigen-presenting cells such as cDC1 at the injection site and in tumor-draining lymph nodes. cDC1s load extracellular vesicles carrying tumor antigen-MHC I complexes and deliver them to conventional DCs in lymph nodes via a synaptosomal-like transport mechanism. These conventional DCs then recognize MHC I-bound tumor antigens through T cell receptors, efficiently cross-activating CD8^+^ T cells. L17-F05-LNP-mRNA, when administered via subcutaneous injection, can specifically target and activate lymph node macrophages, inducing them to upregulate the secretion of cytokines and chemokines such as IFN-β, CCL2, and CXCL10. This enhances the recruitment and activation of antigen-presenting cells and effector T cells. Ultimately, activated CD8^+^ T cells differentiate into effector cytotoxic T cells, migrate to the tumor site, and mediate tumor cell killing, leading to cancer cell death.

Engineering LNPs through chemical modification and affinity-ligand-mediated surface functionalization enables precise targeted delivery of mRNA (128). Zeng et al. synthesized cholesterol-derived mannosyl peptides (CPSM) and cholesterol derivatives modified with mannose (CM), which were used to prepare mannose-modified LNPs. These LNPs demonstrated exceptional mRNA transfection efficiency in dendritic cells and mouse models. Compared to commercial ALC-LNP, CPSM-LNP and CM/CPSM-LNP exhibit significantly greater accumulation in lymph nodes (129). Kim et al. significantly enhanced the targeted delivery of tumor mRNA vaccines via LNPs by introducing PD-L1-binding peptides. A copper-free click reaction covalently linked PD-L1-binding D-peptides to PEGylated lipids, which were then directly integrated into the standard LNP preparation workflow. Optimized Pep LNPs exhibit potent interaction with PD-L1 protein, enabling specific delivery of mRNA to PD-L1-overexpressing tumor cells. This induces autophagy-mediated immunogenic cell death in PTEN-deficient triple-negative breast cancer models, triggering robust antitumor immune responses. This approach provides a tumor-specific therapeutic strategy for PTEN mRNA delivery (130). Papp et al. similarly developed a surface CD47-modified LNP platform (CD47/LNP-mRNA), which significantly reduced the uptake of LNP-mRNA in the mononuclear phagocyte system and liver. This platform enhances targeting efficiency by combining antibodies that target endothelial cells, T cells, and hematopoietic stem cells, achieving three times the efficacy of unmodified LNPs. Consequently, LNPs modified with CD47 enhance targeted delivery to hematopoietic stem cells (HSCs) while reducing off-target effects, providing an effective novel mRNA delivery platform for HSC depletion strategies prior to bone marrow transplantation (131) (**Figure 3**). In summary, LNP surface modification exhibits significant antitumor effects in tumor mRNA vaccines, making it suitable for precise targeting and enhancing specific immune responses. Future research can further explore the development of more efficient and safer tumor mRNA vaccines (125, 127, 129).

## 6 Key obstacles and cutting-edge challenges in the clinical application of mRNA vaccines

Optimization of delivery systems has provided technical support for the precise application of mRNA vaccines. However, their translation from laboratory research to clinical practice still faces multiple challenges. mRNA molecules are composed of nucleotides and carry a negative charge due to their phosphate backbone. This negative charge makes mRNA molecules susceptible to electrostatic repulsion in the extracellular environment (132, 133). Due to this characteristic, mRNA is typically degraded by enzymes specialized in breaking down RNA molecules. In particular, extracellular ribonucleases recognize mRNA vaccines delivered to target cells as foreign RNA and rapidly degrade them (36, 134, 135). Therefore, developing safe and effective delivery vehicles for targeting specific cells has become a critical challenge in this field. Liposomes have become a commonly used delivery vehicle for mRNA vaccines, with their safety and efficacy widely recognized. However, recent studies suggest that liposomes may pose some degree of toxicity when delivering mRNA in the human body, indicating at least that they are not entirely risk-free (136, 137). Although studies have confirmed that, exosomes (138), cationic peptide DP7 (139), serving as a medium has proven effective. However, they have yet to undergo comprehensive clinical trials, particularly in the context of immune suppression within the tumor microenvironment. The inherent immunogenicity of LNPs may trigger an excessive immune response or premature clearance, thereby limiting the vaccine’s efficacy. Secondly, the route of administration determines mRNA distribution and impacts vaccine efficacy (140). Intradermal and subcutaneous injections of mRNA are readily processed by local antigen-presenting cells, potentially causing a more pronounced local injection site reaction. Through systematic review and analysis of extensive literature, this study identifies numerous similar challenges in the development and application of therapeutic mRNA vaccines for lung cancer and the widely deployed COVID-19 mRNA vaccines. These challenges may represent key issues common to the application of mRNA vaccines across different disease areas, necessitating further research and resolution. As vaccines designed using mRNA technology, both the lung cancer mRNA therapeutic vaccine and the COVID-19 mRNA vaccine may share similar limitations regarding delivery stability, sustained immunogenicity, and potential side effects. Studies have confirmed that even when administered at doses up to 10 times higher, intranasal administration of COVID-19 self-amplifying mRNA vaccines still elicits a relatively low immune response (141). It is speculated that mRNA vaccines for lung cancer administered via the nasal mucosa may also exhibit suboptimal immune responses. Furthermore, the lung cancer tumor microenvironment contains multiple bioactive components, including MDSCs, circulating tumor cells, Tregs, tumor-associated fibroblasts, vascular endothelial growth factor, and transforming growth factor. These components may suppress the expression of MHC molecule complexes on tumor cell surfaces, thereby hindering the immune system—particularly CD8^+^ T cells—from recognizing tumor-specific antigens presented on these cells. This significantly diminishes the therapeutic efficacy of mRNA vaccines (68). Furthermore, while long-term immune memory induced by mRNA vaccines forms the foundation for achieving clinical cure, persistent immune surveillance acts as a double-edged sword in a highly heterogeneous disease like lung cancer, potentially driving subsequent immunoediting under selective pressure (142, 143). In the early stages of evolution, T cell-mediated killing of immunogenic cancer cells can temporarily reduce tumor heterogeneity within immune-evading tumors through clonal selection. However, subclones that manage to evade the immune response evolve toward enhanced immune resistance, dampening the effect of immunoediting and fueling a rebound in intratumor heterogeneity that ultimately blunts the overall antitumor immune response (144). Thus, this mechanism highlights how long-term immune surveillance can ultimately foster the evolution of more malignant, highly heterogeneous clonal lineages. Additionally, mRNA vaccines face significant challenges in the selection and long-distance transportation preservation of raw materials (plasmids, lipids, nucleotides, and enzymes). Given the inherent instability of mRNA, the refinement of cold chain technology, the stability and safety of encapsulation materials, and quality control throughout the production process are emerging as key areas for future optimization (13). In a head-to-head comparison between mRNA-1273 and BNT162b2 among males aged 18–25 years, mRNA-1273 vaccination was associated with a higher risk of myocarditis (adjusted incidence rate ratio, 1.43; 95% CI: 0.88-2.34), though this difference did not reach statistical significance (145). However, it remains unclear whether this risk is equally present in patients who have received mRNA vaccines for lung cancer, particularly those who have undergone immune checkpoint inhibitor therapy or chemotherapy and have compromised immune function. Therefore, accelerating the clinical trials of mRNA vaccines for lung cancer is of great significance.

In recent years, the widespread application of mRNA vaccines in lung cancer treatment has remained constrained. Research reports on the safety and stability of LNP-based mRNA vaccines remain scarce, which has to some extent impacted vaccine accessibility and scalability (55). In other words, achieving widespread vaccination remains a distant goal. Therefore, the pressing issue now is how to generate the critical momentum needed to advance clinical trials.

## 7 Conclusion

This review takes mRNA vaccines as reprogrammers of the tumor microenvironment as its central perspective and systematically constructs a theoretical framework spanning from target screening to clinical translation. Antigen screening defines the targets of remodeling, microenvironmental regulation elucidates the mechanisms of remodeling, clinical studies validate the therapeutic efficacy of remodeling, and the optimization of delivery systems ensures the realization of precise remodeling. This perspective may help drive a paradigm shift in mRNA vaccines from a therapeutic modality to a systematic strategy for immune microenvironment remodeling. Nevertheless, the further refinement of this theoretical framework and the full realization of its clinical value still depend on a sustained and in-depth understanding of the complexity of the lung cancer tumor microenvironment. The tumor microenvironment of lung cancer is a highly complex and dynamically changing system, whose regulatory mechanisms involve multiple cell types, molecular signaling pathways, and immune responses. Despite significant advances in understanding the regulatory mechanisms of the tumor microenvironment, precisely modulating this environment to influence tumor progression and metastasis remains a formidable challenge. mRNA vaccines can remodel multiple tumor-associated immune cell populations, including tumor-associated regulatory T cells and MDSCs, by encoding various TAAs. Through signaling pathways such as MYD88 or TRIF, they further activate key factors including NF-κB, CXCL9, CXCL10, and IL-12, thereby reshaping the tumor microenvironment and enhancing the overall antitumor immune response. However, research demonstrates that the TME is a complex and dynamic ecosystem where multiple cellular components—including tumor cells, immune cells, and supporting cells such as fibroblasts, stromal cells, and endothelial cells—coexist. This environment is characterized by intricate interactions among various metabolites and cytokines. Such high heterogeneity poses significant challenges for the effective development of mRNA vaccines (146). Ye et al. stratified low-grade gliomas into three distinct subtypes and proposed tailored therapeutic strategies accordingly: the immune-inflammatory subtype (IS3) is amenable to mRNA monotherapy; the immunosuppressive subtype (IS2) warrants combination with ICIs to overcome checkpoint-mediated resistance; and the immune-desert subtype (IS1) requires prior or co-stimulation to recruit lymphocytes before a vaccine response can be elicited (147). Meanwhile, emerging evidence suggests that mRNA vaccines can induce neoantigen-specific T-cell activity even in non-inflamed tumors characterized primarily by immune-excluded or immune-desert phenotypes (148). At the same time, our current understanding of how mRNA vaccines precisely regulate immune factors and the synergistic mechanisms of their delivery systems remains incomplete. Although some preclinical and clinical studies have evaluated the potential value of mRNA vaccines in lung cancer treatment, overall optimization progress has been relatively slow, and breakthrough results remain limited. This is closely related to the inherent limitations of mRNA therapeutic vaccines for lung cancer, such as the high antigen heterogeneity and the complexity of immune escape mechanisms.

To achieve a systematic breakthrough in overcoming the aforementioned bottlenecks, future research must adopt a multidimensional integrated strategy, particularly in key areas such as novel specific antigen screening, innovative delivery carrier systems, and tumor microenvironment regulation (149, 150). Specifically, by integrating tumor multi-omics data (genome, transcriptome, and proteome) through high-throughput sequencing combined with AI algorithms, novel TSA and their associated immune epitopes can be precisely identified. This enables the development of scientifically sound solutions based on optimized sequence structures. Improvements to delivery systems are critical for the clinical application of mRNA vaccines. Future efforts can focus on two key directions: optimizing LNPs through engineered approaches and exploring the innate immune regulatory advantages of exosome carriers to achieve precise delivery to tumor targets (151, 152). Secondly, in terms of regulating the immune tumor microenvironment, the combination of mRNA therapeutic vaccines with immune checkpoint inhibitors (such as PD-1/PD-L1 antibodies) or small-molecule immunometabolic modulators (such as IDO inhibitors) has demonstrated significant synergistic effects in some studies (153), such combination therapies are emerging as a significant trend in lung cancer treatment. Beyond these exogenous combination strategies, leveraging ferroptosis has emerged as a cutting-edge approach to enhance the immunogenicity of cancer vaccines. Mechanistically, tumor cells undergoing early-stage ferroptosis can effectively trigger immunogenic cell death and release damage-associated molecular patterns (DAMPs), such as adenosine triphosphate (ATP) and high mobility group box 1 (HMGB1), thereby eliciting a potent and protective anti-tumor immune response. This mechanism has been validated in pancreatic cancer research: among the three subtypes (FS1-3) identified via ferroptosis-related gene expression profiles, the FS3 subtype, which is associated with the most favorable prognosis, exhibits a marked downregulation of HMGB1. This finding suggests that for specific molecular subtypes lacking endogenous DAMP release, utilizing ferroptosis-associated antigens to induce early ferroptosis in target cells holds promise as a highly effective precision vaccine strategy (154). Furthermore, a similar mechanism was observed in a study on a DC vaccine. This study demonstrated that inducing ferroptosis in tumor cells exerts a potent “endogenous adjuvant” effect through the release of DAMPs such as calreticulin and ATP, significantly enhancing the infiltration of CD8^+^ T cells into the TME (155). Therefore, in the design of mRNA vaccines, synergistically inducing ferroptosis in target tumor cells could substantially amplify the immune cascade and anti-tumor efficacy. Furthermore, the design of neoantigen mRNA vaccines holds promise for iterative upgrades of specific therapeutic vaccines through dynamic monitoring of tumor mutation profiles, thereby better countering tumor immune evasion mechanisms.

In summary, integrating multidimensional data and applying AI-driven mRNA design for personalized vaccine development holds promise for establishing an iteratively optimized therapeutic mRNA vaccine system for lung cancer. This approach lays the groundwork for breakthrough progress in mRNA vaccines for lung cancer treatment.
